# Recent Advances in Paper-Based Sensors

**DOI:** 10.3390/s120911505

**Published:** 2012-08-24

**Authors:** Devi D. Liana, Burkhard Raguse, J. Justin Gooding, Edith Chow

**Affiliations:** 1 CSIRO Materials Science and Engineering, P.O. Box 218, Lindfield, NSW 2070, Australia; E-Mails: d.liana@student.unsw.edu.au (D.L.); burkhard.raguse@csiro.au (B.R.); 2 School of Chemistry, The University of New South Wales, Sydney, NSW 2052, Australia; E-Mail: justin.gooding@unsw.edu.au

**Keywords:** paper, sensor, device, microfluidics, lab-on-a-chip, diagnostics, analytical

## Abstract

Paper-based sensors are a new alternative technology for fabricating simple, low-cost, portable and disposable analytical devices for many application areas including clinical diagnosis, food quality control and environmental monitoring. The unique properties of paper which allow passive liquid transport and compatibility with chemicals/biochemicals are the main advantages of using paper as a sensing platform. Depending on the main goal to be achieved in paper-based sensors, the fabrication methods and the analysis techniques can be tuned to fulfill the needs of the end-user. Current paper-based sensors are focused on microfluidic delivery of solution to the detection site whereas more advanced designs involve complex 3-D geometries based on the same microfluidic principles. Although paper-based sensors are very promising, they still suffer from certain limitations such as accuracy and sensitivity. However, it is anticipated that in the future, with advances in fabrication and analytical techniques, that there will be more new and innovative developments in paper-based sensors. These sensors could better meet the current objectives of a viable low-cost and portable device in addition to offering high sensitivity and selectivity, and multiple analyte discrimination. This paper is a review of recent advances in paper-based sensors and covers the following topics: existing fabrication techniques, analytical methods and application areas. Finally, the present challenges and future outlooks are discussed.

## 1. Introduction

Paper is a well-known material for writing, printing, drawing and packaging. The potential utility of paper beyond these simple and traditional means stems from its physical properties. It is a highly sophisticated material as it can be made thin, lightweight and flexible depending on its pulp processing. The main constituent of paper is cellulose fibre, and this can be highly attractive for certain applications as it allows liquid to penetrate within its hydrophilic fibre matrix without the need of an active pump or external source [1]. Moreover, cellulose fibres can be functionalised, thus changing properties such as hydrophilicity, if desired, as well as its permeability and reactivity [2].

Recently, paper has drawn much interest as a potential material for sensors and devices in analytical and clinical chemistry because of its versatility, high abundance and low cost [1,3–6]. These analytical devices can be integrated in a manner that is flexible, portable, disposable and easy to operate. Following the invention of paper chromatography in the early 20th century [7,8], diagnostic devices based on paper began to emerge.

In 1956, the first paper device for the semi-quantitative detection of glucose in urine was demonstrated [8], that further developed into immunochromatographic paper test strips (also known as lateral flow or dipstick tests), with the pregnancy test kit being a well-known example [9]. These immunoassays consist of a strip of paper with a sample pad (for introduction of the sample), reagent pad (containing antibodies conjugated to a signal indicator which are specific to the target antigen) and a test line (capture antibodies immobilised on the surface). When the sample is introduced at the sample pad, it migrates along the paper strip via capillary forces, where the presence of the antigen in the sample binds to the signal antibody. The formed antigen/signal antibody continues to flow along the paper strip where it is subsequently captured at the surface by the capture antibody to give a positive result. The signal indicator is typically coloured latex microspheres or gold nanoparticles [10]. Although reliable, these simple and low cost devices are generally limited in providing a qualitative “yes/no” type of detection.

The last few years has seen a shift in focus from basic design concepts to more advanced fabrication and patterning techniques in order to obtain more accurate and quantitative results. Whitesides and co-workers [1,11] introduced the idea of fabricating microfluidic channels on paper (μPADs) for multiplex analyte detection. The detection method is based on colorimetry which measures colour intensity in relation to the concentration of the analyte. Since then, many new areas of fabrication and exploration have opened up, such as in paper-cut microfluidic devices [12–15] and microfluidic separation devices where chromatographic separation of mixtures takes place as the solution moves up the paper [16–18]. These new research avenues have resulted in sensors that can be analysed by techniques other than colorimetry [1,11,19–23], such as by electrochemical [24–26], chemiluminescence [7], electrochemiluminescence [27] and electrical [28,29] methods. These techniques have their own advantages and disadvantages in terms of sensitivity, simplicity and cost-effectiveness.

Due to the development of paper-based microfluidics in the past few years, paper has become a promising platform for lab-on-a-chip devices in which large-scale and complicated laboratory tests could be performed. Moreover, they allow for portable, on-site real-time detection which is crucial in many applications such as in the clinical, food and environmental sectors where simple and practical analytical devices are highly needed. With the spiralling costs of health care, there is an increasing demand for point-of-care (POC) diagnostics to obtain more rapid test results, a challenge which could potentially be met by paper-based sensors. The current review will introduce the paper types commonly used for sensing, discuss existing fabrication and analysis techniques, and application areas for these sensors. Present challenges that need to be addressed in order for paper-based sensors to reach their full potential will be discussed, as well as future outlooks.

## 2. Development of Paper-Based Sensors

### 2.1. Paper Choices

There are a variety of paper materials available to the user, although the choice is based mainly on the fabrication steps required in developing a device and also on the specific application area. In the development of sensors and microfluidic technologies, filter paper has seen widespread use in recent years for producing paper-based sensors due to its wicking ability [1,13,30,31]. In particular, the Whatman^®^ cellulose range is popular with the important parameters differentiating the filter paper types being porosity, particle retention and flow rate. Many groups [1,7,13,16,22,30,32–36] used Whatman^®^ filter paper No. 1 in their work which is a standard grade filter paper with medium retention and flow rate. However, in order to increase liquid penetration, Li *et al.* [31] used Whatman^®^ No. 4 filter paper and coated it with a cellulose hydrophobisation agent as a base for etch printing of hydrophilic channels. This type of filter paper has a larger pore size than the standard grade and was chosen because swelling of the cellulose fibres by the solvent can restrict the capillary pores and thus hinder liquid penetration.

Although filter paper is widely used, it does not always possess the desired physical characteristics so other types of paper or paper modifications have been explored. For instance, hydrophobic nitrocellulose membranes exhibit a high degree of non-specific binding towards biomolecules and are suitable for immobilisation of enzymes [22], proteins [13] and DNA [37]. Lu *et al.* [38,39] explored the use of a nitrocellulose membrane as the substrate in constructing a paper-based sensor, first by forming a wax barrier on the membrane by printing and heating, followed by deposition of an enzyme for a colorimetric assay. Although, nitrocellulose membranes are smooth and have a reasonably uniform pore size (0.45 μm), which results in a more stable and reproducible liquid flow within the paper, the wax penetration is slow compared to filter paper. Another avenue for exploration is the use of chemically modified cellulose fibres. There exist commercially available ion-exchange cellulose papers and composite papers consisting of cellulose and polyester [26].

Instead of using filter paper as the main material to create paper-based sensing devices, other types of paper such as glossy paper have been reported as a suitable platform in sensor technologies. Glossy paper is a flexible substrate made of cellulose fibre blended with an inorganic filler. Arena *et al.* [28] used glossy paper for developing a flexible paper-based sensor for the detection of ethanol using indium tin oxide nanoparticulate powder as a sensing material and multi-walled carbon nanotubes as electrodes. Due to the non-degradability and relatively smooth surface of glossy paper, it is a good substitute for filter paper especially when modifying nanomaterials onto a surface rather than within the fibre matrix is necessary.

### 2.2. Fabrication and Patterning

In fabricating paper devices, the choice of techniques and materials that meet the criteria of low cost, simplicity and efficient production process need to be considered. There are several techniques and processes involving chemical modification and/or physical deposition that could be used to tune the properties of the paper such that it becomes available for further modification or direct usage in a range of applications [40].

Techniques reported in the literature include photolithography [11,19,20], analogue plotting [21], inkjet printing [41] and etching [22,23,31], plasma treatment [42,43], paper cutting [12,13], wax printing [44–46], flexography printing [47], screen printing [5], and laser treatment [1,48]. Techniques were chosen depending on the type of material used and the type of modification required. Much research is focused on confining the liquid to a specific region on the paper, in what is known as paper-based microfluidics, so first we discuss some of these approaches followed by some other methods to build up the active sensing element.

**Figure 1.**
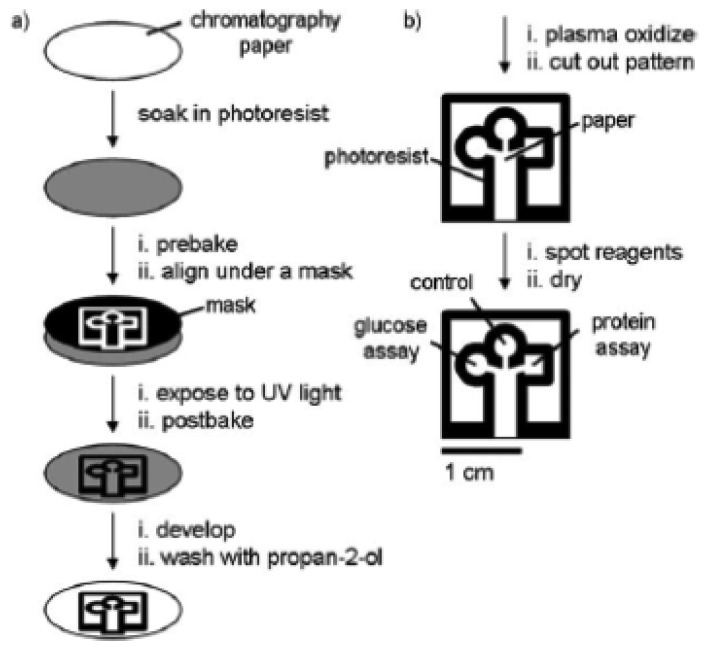
(**a**) Steps involved in fabricating paper with millimetre-sized channels using photolithography and (**b**) spotting of the paper for glucose and protein assays. (Reprinted with permission from Martinez *et al. Angew. Chem. Int. Ed.* **2007**, *46*, 1318–1320. Copyright © 2007 John Wiley & Sons [11]).

In 2007, Martinez *et al.* [11] introduced a lithographic technique to create a microfluidic channel by using a hydrophobic photoresist, SU-8 polymer (Figure 1). The hydrophilic channel defined the liquid penetration pathway as it was confined within the hydrophobic walls. As the liquid was introduced to the hydrophilic channel, it moved through the paper matrix by capillary flow action. A three-branch tree pattern was lithographically patterned on the paper for the reaction site where different reagents were spotted for glucose and protein assays. This work was a major breakthrough that led to significant research growth in this field. It is attractive as it offers a simple and relatively inexpensive alternative to existing technologies and is suitable for portable applications.

Since the pioneering work by Martinez *et al.* [1,11,22] on paper-based microfluidics, alternative approaches have been introduced by other researchers to create a hydrophilic channel confined within a hydrophobic barrier. Physical deposition of patterning agents such as wax [5,35], polydimethylsiloxane [21] and polystyrene [47] have been used to create paper devices by many research groups.

One potential problem with the use of photolithography to create a microfluidic channel is that damage of the photoresist may occur during bending or folding. To overcome the problem introduced by photolithography, Bruzewicz *et al.* [21] demonstrated printing of elastomeric polydimethylsiloxane onto paper using a plotter. This allowed folding of the paper device without destruction of the channel. Moreover, the technique is highly reproducible and uses inexpensive materials, and is thus suited for even the most basic of research laboratories where microfluidic devices with feature sizes of ∼1 mm are adequate.

**Figure 2.**
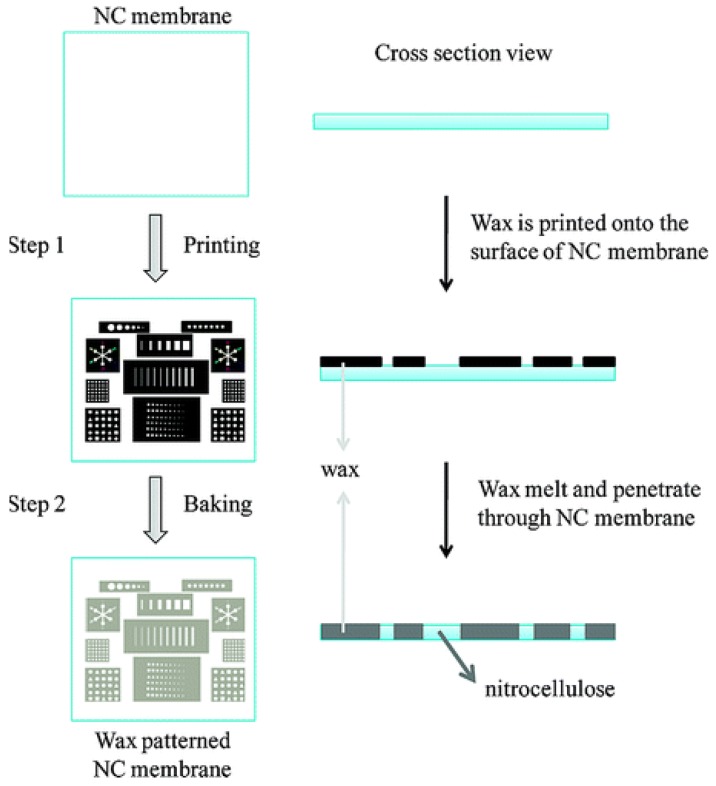
Schematic diagram of printing wax onto a nitrocellulose (NC) membrane. The first step involves patterning the wax onto the membrane using a wax printer and in the second step the membrane is placed in an oven at 125 °C for 5 minutes to melt the wax. (Reprinted with permission from Lu *et al. Anal. Chem.* **2010**, *82*, 329–335. Copyright © 2010 American Chemical Society [38]).

Alternatively, wax printing can be used to create a microfluidic channel as demonstrated through independent studies by Carrilho *et al.* [49] and Lu *et al.* [38,44]. Solid wax patterns can be printed on the surface of paper followed by the use of a heat source such as an oven, hot plate or heat gun to melt the wax (Figure 2). Carrilho *et al.* [49] introduced the wax printing technique as a rapid, inexpensive and efficient process for prototyping a device in under 5 minutes. The technique is preferable for the fabrication of large quantities of sensors because fewer steps are involved in forming the hydrophobic barrier compared to photolithography. The heating process allows the wax to penetrate both vertically and horizontally within the paper matrix. Spreading of the wax vertically through the paper will effectively confine the solution flow to the desired regions of the paper. However, due to the nature of the fibre matrix, the paper tends to align the wax in a horizontal, rather than vertical direction [49]. As a result, the wax spreads faster in the horizontal direction causing a wider line compared to the original width of the applied wax [49]. Therefore, the reproducibility of the fabrication method is highly dependent on the width of the wax line and the heating temperature.

Lu *et al.* [38,44] assessed three different processes of patterning filter paper using wax and these are: (i) painting wax using a wax pen; (ii) employing an inkjet printer followed by wax painting, and (iii) using a wax printer directly to print the wax. Wax painting is relatively straightforward as it only requires drawing the pattern with a wax pen followed by melting. However, the method is not suitable for creating complicated patterns. When complicated pattern design is necessary, the pattern can be printed on paper first and then painted with wax. Alternatively, the need for a wax pen can be eliminated by direct wax printing. Ge *et al.* [50] also used this wax printing technique to pattern and fabricate 3-D origami-based devices which will be discussed later on.

Instead of printing wax on the paper, Songjaroen *et al.* [35] used a wax dipping technique to fabricate the sensors. First, the paper was placed on a glass slide and an iron-shaped mould was attached to the paper using a magnet on the backside of the glass. This was then placed into melted wax for one second, followed by removal of the iron-shaped mould after the paper had cooled. The region where the mould was, defined the hydrophilic test zones. Other methods such as screen printing the wax have been investigated by Dungchai *et al.* [5] which involved rubbing a solid wax through the patterned screen onto a paper that was subsequently melted so that the wax could penetrate through the paper.

Approaches that involve selective removal or modification of the hydrophobic material after deposition have also been investigated in processes such as inkjet etching and plasma treatment. Abe *et al.* [23] formed hydrophilic channels by first hydrophobising paper by soaking it in a toluene solution containing poly(styrene). Subsequently, the hydrophilic region was defined by inkjet deposition of toluene to locally dissolve the poly(styrene) away from selected regions. Alternatively, Li *et al.* [42,43] used plasma treatment to chemically modify the surface. First they immersed filter paper into a hydrophobisation agent, alkyl ketene dimer (AKD), and then the hydrophobic AKD-treated paper was sandwiched between two metal masks and the hydrophilic channel for liquid transport was formed by plasma treatment of the functionalised paper.

**Figure 3.**
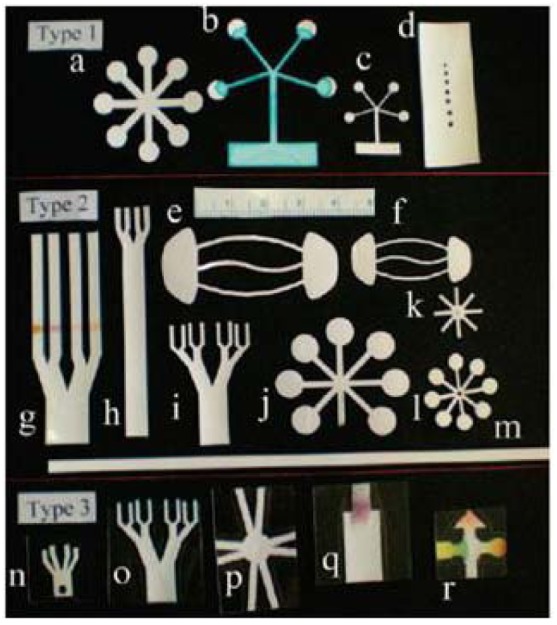
Three types of devices that have been cut into a variety of two dimensional shapes. Type 1 is fabricated by shaping polyester-backed nitrocellulose membrane, Type 2 is formed by capping the nitrocellulose membrane or filter paper with a polyester cover tape and then shaping it and Type 3 is fabricated by first shaping the nitrocellulose membrane or filter paper and then capping both sides with a polyester cover tape and then creating an inlet for the fluid. (Reprinted with permission from Fenton *et al. ACS Appl. Mater. Inter.* **2009**, *1*, 124–129. Copyright © 2009 American Chemical Society [13]).

Not all paper sensing devices require the formation of a hydrophilic channel within a hydrophobic barrier. Wang *et al.* [12] demonstrated a tree-shaped paper for the semi-quantitative detection of protein. The filter paper was cut into a tree-shaped sheet with seven branches (3 mm × 45 mm) and a stem (8 mm × 55 mm). The design allows uniform microfluidic flow along the multiple branches when the stem is placed into a wicking solution and spotted with the sample at the branch. Wang *et al.* [12] have demonstrated that calibration standards and measurements of an unknown can be performed at the same time using this tree structure. In a more defined approach, Fenton *et al.* [13] used a computer controlled X-Y knife plotter to form star, candelabra and other structures with the paper. They sandwiched the paper between vinyl and polyester plastic films in order to prevent evaporation and protect the surface from contamination and dehydration during the assay procedure (Figure 3). Shaping the paper by cutting has proven to be an easier technique compared to photolithography where multiple steps are required.

Depending on the types of analyses to be performed on the paper devices, integration of other elements onto paper devices may be required. For instance, Nie *et al.* [26] and Dungchai *et al.* [24] screen-printed electrodes on the paper for electrochemical analysis. Moreover, Hossain *et al.* [6] explored the use of inkjet printing to deposit three layers of different chemical materials on paper. The three layers consisted of poly(vinylamine) printed directly on top of the paper, silica sol as an intermediate layer, and a top layer containing the enzyme acetylcholinesterase and the chromogenic agent dithiobisnitrobenzoate in Tris buffer. The paper device was used as a solid-phase sensor for detecting neurotoxins where the interaction between the analyte and the top printed layer resulted in a decreased enzyme activity which produced a change in colour intensity.

Other types of modifications include depositing metal nanoparticles, as an alternate material to dye molecules, onto paper as a colour indicator [10,36]. Zhao *et al.* [10] demonstrated a paper-based gold nanoparticle sensor which was used to detect the endonuclease that cleaves DNA, DNase I, as well as a small biomolecule, adenosine.

The microfluidic fabrication and patterning techniques discussed earlier can be used to extend paper as one-dimensional (1-D) and two-dimensional (2-D) lateral flow systems to more complex three-dimensional (3-D) microfluidic devices. The 3-D devices allow rapid distribution of sample in the z-direction and for multiple assay processes to be performed, yet still allowing sample volumes to be kept to a minimum [19]. The principles of 2-D [1,5,14,30,51,52] and 3-D [19,53,54] paper analytical devices have been discussed by many researchers. As detailed earlier, the 2-D microfluidic devices are created by patterning a hydrophobic wall using wax or photoresist on a sheet of filter paper, with movement of liquid driven by capillary force. In order to create a 3-D microfluidic device, 2-D microfluidic devices can be stacked together using alternating layers of patterned paper and double-sided tape as demonstrated by Martinez *et al.* [19]. Holes were laser cut into the tape and filled with cellulose powder [19] or compressed [33] to allow fluids to wick between layers [19]. Such 3-D devices allow microlitre volumes of liquid to be distributed across many detection zones from a single inlet [33].

**Figure 4.**
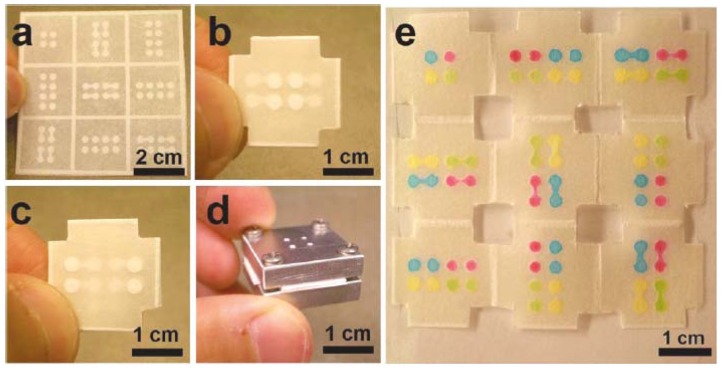
(**a**) Filter paper with photolithography-patterned channels, reservoirs and a folding frame. (**b**) Top layer of the folded paper showing a four- inlet reservoir in the centre of device. (**c**) Bottom view of the paper device. (**d**) The assembled device held together by aluminium housing (**e**) An unfolded paper showing the nine layers of the paper-based microfluidic device after injection of four 1.0 mM solutions of rhodamine 6G (red), erioglaucine (blue), tetrazine (yellow) and a 1:10 mixture of erioglaucine and tetrazine (green). (Reprinted with permission from Liu *et al. J. Am. Chem. Soc.* **2011**, *133*, 17564–17566. Copyright © 2011 American Chemical Society [54]).

3-D paper microfluidic devices were further developed with only a single sheet of patterned paper and folded based on the origami principle [50,54,55]. The system successfully eliminates the need of adhesive tape and laser cutting. The 3-D origami-based devices can be generated by creating a hydrophobic barrier using photoresist [54] or by wax printing [50]. Liu *et al.* [54] fabricated a paper-based microfluidic device by patterning a piece of filter paper using conventional lithographic techniques to create channels and detection reservoirs housed within a frame. The folded paper was then trimmed at the edges and clamped together with aluminium plates which had four holes in the centre to allow solution to be injected (Figure 4).

Recently, Lewis *et al.* [56] developed an efficient and high throughput method for fabricating large quantities of 3-D paper-based microfluidic devices. The fabrication process involved patterning the entire sheet of paper by wax printing and combining the layers of patterned paper using a spray adhesive. This simple assembly method is capable of producing 200–300 devices simultaneously and is rapid as it relies on alignment of paper by the sheet edges rather than by features on the device.

## 3. Quantitative Analysis

Paper-based sensors offer users the possibility to produce simple, mass-scalable devices at an affordable cost. However, in order to create an analytical device, suitable transduction methods are necessary which may require additional reagents, materials and instrumentation at further cost and complexity. In order to maintain simplicity, affordability and portability, low power techniques such as optical and electrochemical methods are well suited as transducers. The five most commonly reported techniques for quantitative analysis on paper are colorimetric [11–13,20,23,57], electrochemical [5,18,24–26,55,58], chemiluminescence [7,50], electrochemiluminescence [27,53] and electrical conductivity [28,29].

### 3.1. Colorimetric Detection

Colorimetric detection has been widely used in paper-based microfluidic devices when a “yes/no” answer or semi-quantitative result is sufficient for analysis. This is perhaps the simplest of techniques as a change in colour may be visualised by eye due to an enzymatic or chemical interaction. Martinez *et al.* [11] first demonstrated paper-based microfluidic devices for the colorimetric detection of glucose and protein. The investigation was based on a change in colour when introduction of the sample filled the reaction zone.

For the glucose assay, a positive result was observed when the colour shifted from clear to brown which was mainly due to the enzymatic oxidation of iodide to iodine whereas for the protein assay, a positive result was indicated by a colour change of tetrabromophenol blue from yellow to blue.

Recently, Ratnarathorn *et al.* [57] demonstrated the colorimetric detection of copper using silver nanoparticles (AgNP) on paper devices. The paper-based devices were fabricated by immobilising AgNPs functionalised with homocysteine (Hcy) and dithiothreitol (DTT) in the test zone. Once the sensor was fabricated, the copper solution was dropped onto the loading zone and the liquid was transported to the test zone. A colour change was observed upon copper-induced aggregation of Hcy-DTT-AgNPs through binding of copper to the carboxyl and amino functional groups on Hcy and DTT. Here the limit of detection by the naked eye is 7.8 nM.

Although the above analytical techniques do not require additional instrumentation, they are not as accurate compared to techniques such as UV-visible spectroscopy and atomic absorption spectroscopy. This is mainly because the colour and intensity interpretation by eye is different for each individual, ambient light condition, as well as the condition of the paper substrate (dry or wet).

**Figure 5.**
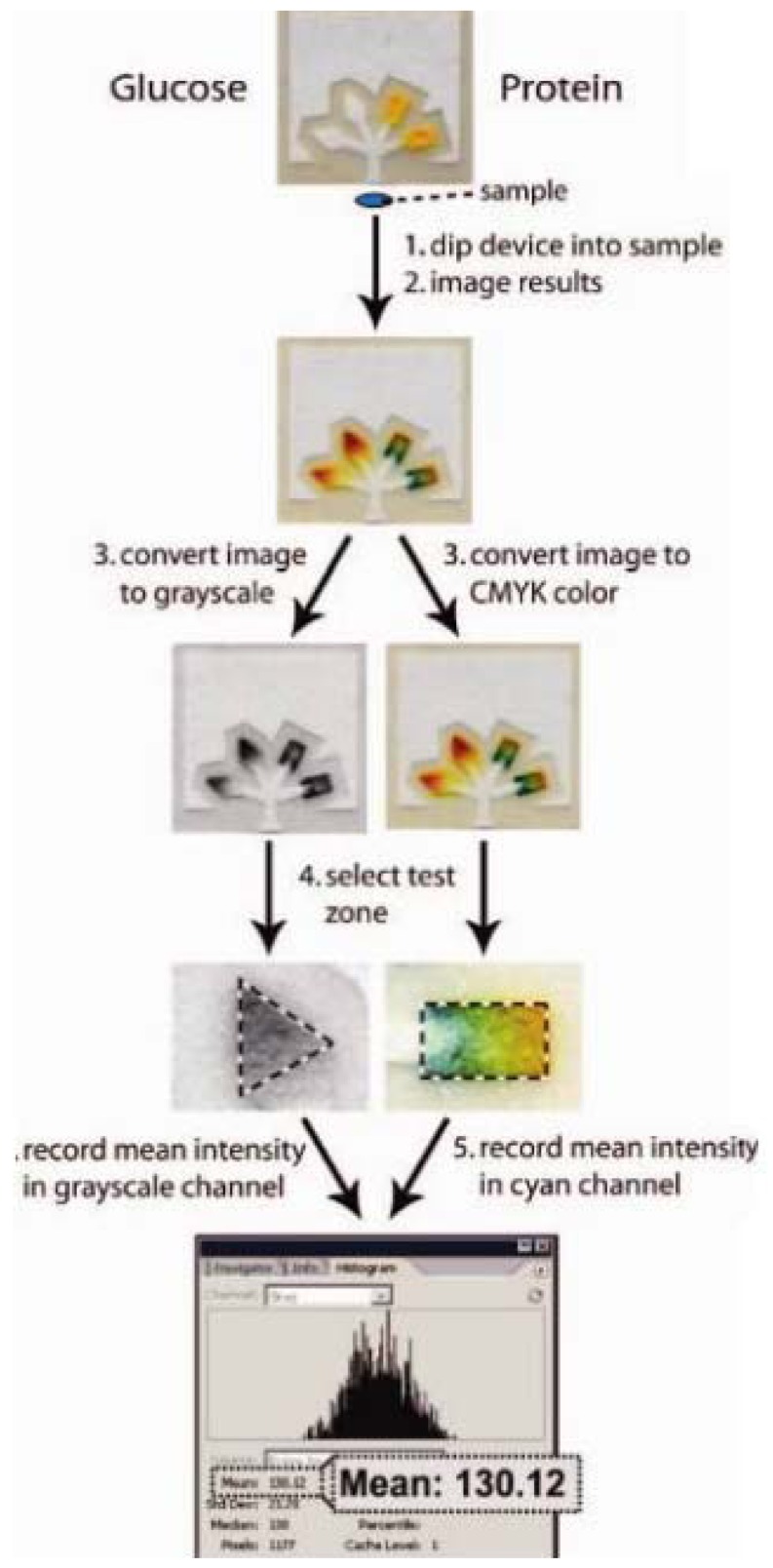
Procedure for quantifying the amount of glucose and protein in a urine sample using Adobe^®^ Photoshop^®^ by converting the images into greyscale (for glucose assay analysis) and CMYK colour (for protein assay analysis) to obtain the mean pixel values in the test zones which correlates to the concentration of the analytes in the sample. (Reprinted with permission from Martinez *et al. Anal. Chem.* **2008**, *80*, 3699–3707. Copyright © 2008 American Chemical Society [22]).

In order to improve the accuracy and sensitivity of the method, Martinez *et al.* [22] measured visible colour changes by using a camera phone or scanner (Figure 5). The image was captured and transferred to a computer and the colour was interpreted using imaging software where a pixel value is related to the concentration of the analyte. By using this system, data analysis could be easily interpreted.

### 3.2. Electrochemical Detection

Electrochemical techniques often require a three-electrode system, that is, a counter electrode, reference electrode and working electrode. In creating a paper-based electrochemical sensor, a three-electrode system is replicated on paper. This means that such a sensor could replace the traditional electroanalytical method where expensive solid state electrodes are required. As discussed earlier in Section 2.2, in order to develop such an electrochemical sensor, an additional step in the fabrication process is the deposition of electrodes, usually in the form of conductive inks, on the paper. Additionally, in order to perform voltammetric experiments a potentiostat is necessary which is low in power and can be made portable.

**Figure 6.**
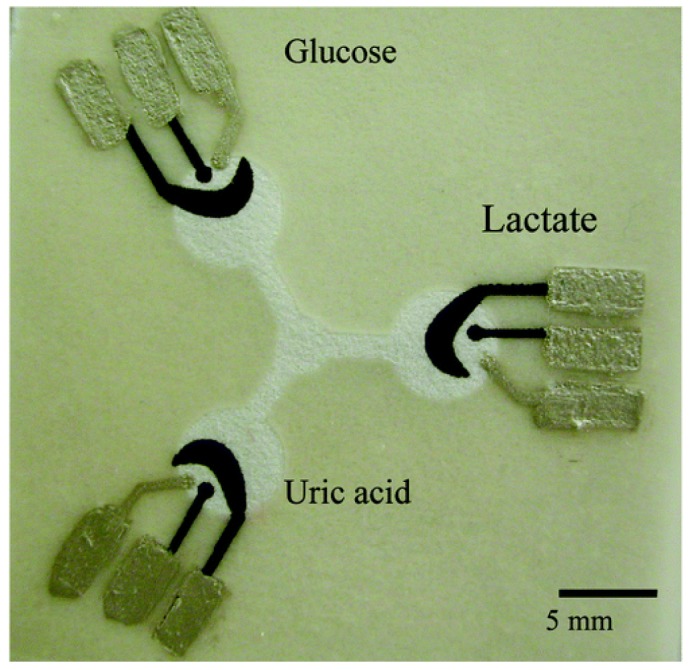
Paper-based microfluidic devices with three electrode system (counter and working electrodes consisting of carbon, and reference electrode consisting of silver/silver chloride) for the electrochemical analysis of glucose, lactate and uric acid. Each of the working electrodes were individually spotted with glucose oxidase, lactate oxidase and uric acid. (Reprinted with permission from Dungchai *et al. Anal. Chem.* **2009**, *81*, 5821–5826. Copyright © 2009 American Chemical Society [24]).

A wide range of inks can be used to produce electrodes on various substrates including paper. The common materials used for creating the electrodes are carbon inks for fabricating working and counter electrodes while silver/silver chloride ink is used for the reference electrode [24,26]. Dungchai *et al.* [24] combined electrochemical analysis for the detection of glucose, lactate and uric acid on paper-based electrodes for the first time. The conductive electrode pads were screen-printed in the hydrophilic region of the paper where enzymes were subsequently spotted. The electrode design is shown in Figure 6. The sample solution was deposited on the centre of the paper and it flowed to the reaction sites that contained the three-electrode system. The analysis was performed using chronoamperometry at the optimal potential for hydrogen peroxide production. The hydrogen peroxide arises from the oxidase enzymes which catalyses the oxidation of the enzyme substrate such as glucose, uric acid and lactate while reducing oxygen to hydrogen peroxide. Moreover, they also performed electrochemical analysis for the detection of gold and iron [30]. The electrochemical analysis was carried out using cyclic voltammetry and square wave voltammetry experiments.

Nie *et al.* [25] performed an electrochemical analysis of glucose and alcohol on paper-based microfluidic devices using a commercial hand-held glucometer as a reader. The paper devices consisted of microfluidic channels, electrodes and electrical interconnections fabricated onto the paper substrate using wax printing and screen printing. The four electrodes which were a working electrode, a counter electrode and two internal reference electrodes were made of graphite ink while the electronic wires were patterned using silver ink. The analysis was conducted by pipetting a sample aliquot into the detection zone of the paper sensor which contained the chemical reagent for each assay. Once the sample was introduced, the liquid sample wicked to the sensing region and the glucometer initiated the amperometric measurement.

Recently, Lankelma *et al.* [58] created a new design for a paper-based electrochemical system for flow injection analysis. The system comprised a nitrocellulose sheet with one end in contact with an upper buffer reservoir and the other end in contact with a long filter paper strip immersed in a lower reservoir sink. Continuous and constant flow of buffer solution was achieved by gravity-driven capillary wicking. The success of this paper-based sensor was demonstrated by detecting glucose in urine. The glucose oxidase was strategically immobilised on the downstream end of a nitrocellulose pad which was in contact with a platinum working electrode. This allowed oxidation of potentially interfering redox species to occur upstream of glucose oxidase, resulting in temporal and spatial resolution of the analyte and interfering species.

Other types of electrode materials that have been used as a paper sensing platform include gold which was deposited through a shadow mask using a sputtering technique as demonstrated by Shiroma *et al.* [18].

### 3.3. Electrical Conductivity

Electrical conductivity measurements involve measuring changes in the conductance of a material in the presence of a chemical species. Despite its simplicity and low power requirements, there have only been a few publications [28,29] regarding this technique for paper-based sensing and these have been limited to applications in the gas-phase.

Arena *et al.* [28] investigated the response of a paper-based sensor for ethanol detection. The sensor was fabricated by integrating multi-walled carbon nanotube electrodes with an indium tin oxide nanoparticulate powder sensing layer on paper. The addition of poly-diallyldimethylammonium chloride as the binder promoted the adhesion of the indium tin oxide nanoparticulate powder to the glossy paper substrate. Subsequent analysis of ethanol was carried out in air by measuring the current upon applying triangular applied voltage.

Steffens *et al.* [29] developed a low cost gas sensor using graphite interdigitated electrodes coated with a thin sensing layer of doped-polyaniline. Changes in conductivity were observed in response to the presence of nitrogen gas.

### 3.4. Chemiluminescent and Electrochemiluminescent Detection

Chemiluminescence and electrochemiluminescence are analysis methods that have also been successfully performed on paper-based sensing devices. These techniques have gained attention due to their simplicity, low cost and high sensitivity. Yu *et al.* [7] demonstrated a microfluidic paper-based device for the detection of glucose and uric acid based on the chemiluminescence reaction between a rhodamine derivative and hydrogen peroxide. The paper device consisted of an injection hole for introducing the sample to the filter paper, two bioactive channels where the enzymes (glucose oxidase and urate oxidase) were immobilised and two chemiluminescent detection areas containing the rhodamine derivatives. All the components were integrated between two water impermeable single-sided adhesive tapes. Interaction of a sample containing glucose or uric acid with the oxidase enzymes, produced hydrogen peroxide, which in turn interacted with the rhodamine derivatives to produce chemiluminescence. The quantitative analysis is based on the peak intensity of the emitted light which is proportional to the concentration of the analyte.

**Figure 7.**
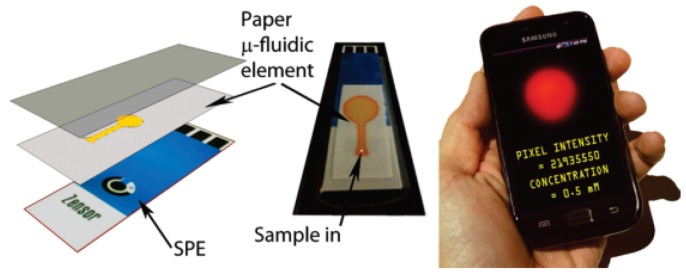
Fabrication and analysis process of paper-based microfluidic electrochemiluminescence sensor. The hydrophilic region of the paper (in yellow) was spotted with (Ru(bpy)_3_^2+^) solution and then aligned onto the screen-printed electrode (SPE) by laminating with a transparent plastic. A small amount of sample was introduced through a small hole in the plastic at the base of the channel and the camera phone was placed close to the paper sensor, upon which a potential of 1.25 V was applied, and the emission was captured and analysed. (Reprinted with permission from Delaney *et al. Anal. Chem.* **2011**, *83*, 1300–1306. Copyright © 2011 American Chemical Society [27]).

Combining chemiluminescence with electrochemical methods has resulted in the analysis technique known as electrochemiluminescence. Whilst chemiluminescence alone has proven to be effective, the combination of the two techniques provides better selectivity and increases the dynamic concentration range [27,53]. Delaney *et al.* constructed paper microfluidic devices [27] based on this analysis method. The fluidic channel was loaded with tris(2,2′-bipyridyl)ruthenium(II) (Ru(bpy)_3_^2+^) before the paper was laminated onto the face of a screen-printed electrode. The reaction was conducted by stepping the potential positively to 1.25 V to oxidise the ruthenium complex. Electrochemiluminescence emission was generated through the interaction between the oxidised form of the ruthenium complex with its co-reactant 2-(dibutylamino)-ethanol or nicotinamide adenine dinucleotide. The added attractiveness of this technique is that it uses a camera phone to detect the electrochemiluminescence emission where the intensity of the red pixel can be used to determine the concentration of the analyte (Figure 7).

The analysis methods as applied to paper-based sensors are summarised in Table 1 in terms of their ability to provide a quantitative analysis result.

## 4. Application of Paper-Based Sensors

Paper sensing devices are a promising platform that, in principle, can be applied across a range of application areas such as in health diagnostics [5,7,11,19,20,23–25,31,33,42,44,58–64], environmental monitoring [26,30,58,65,66] and food quality control [25,58,67]. The paper itself has many advantages compared to plastic and glass substrates because it is low cost, disposable, abundant and easy to transport. To date, researchers have focused on developing paper-based sensors with less complicated fabrication techniques and operation such that it can be used in developing world applications where simple and easy to operate devices are highly desirable.

### 4.1. Health Diagnostics

Paper sensing devices are attractive as potential lab-on-a-chip (LOC) devices. The ideas of LOC are mainly to minimise the scale of laboratory tests and allow portable POC diagnostics and on-site detection [3]. Current LOC products are still considered relatively expensive for users in the developing world [68] because of complex fabrication processes that involve creating a channel, pumps and valves on plastic or glass, as well as the requirements of fabrication in a lithography-based clean room setting [35]. Much effort has been directed to overcome such limitations by eliminating the need for active pumps. Several existing LOC tests use porous materials such as membranes, fleeces and meshes to allow passive transport of liquid [69]. These LOC products have several advantages such as being low in cost, requiring no external power equipment for operation and they can be fabricated by rapid prototyping which is suitable for POC diagnostics in the developing world. In order to further decrease the production cost, researchers have shifted their attention to paper following the development of paper-based microfluidic devices. Since paper fulfils the primary criteria of diagnostic devices (low cost), it will create wider opportunities for LOC device usage in the developing world.

To date, several studies involving paper-based sensors have been conducted using real samples such as urine, saliva and blood for the detection of various types of analytes for clinical diagnosis. Analytes such as glucose [5,7,11,19,20,23,44,58], uric acid [24,42,61], protein [11,20,33], lactate [59], nitrate [20,31,33,42], ketone [20,33], cholesterol [25] and nucleic acid [60] have been studied.

In the diagnosis of diseases, early detection is vital in order for the treatment to be successful. By using cheap paper-based microfluidic devices, it makes diagnosis accessible to all, and for a rapid result to be obtained with facile device operation. An example of this application is the work of Ge *et al.* [50]. Based on such a simple and well established concept of paper folding, the group has shown that chemiluminescence detection of four cancer biomarkers ((r-fetoprotein (AFP), carcinoma antigen 153 (CA153), carcinoma antigen 199 (CA199), and carcinoembryonic antigen (CEA)) in whole blood samples can be performed in a single run without the need for multiple washing steps.

Detecting blood type (ABO) is also crucial for many medical procedures. However, there is no convenient and low cost disposable test available for direct analysis of blood samples. Khan *et al.* [62] found paper as an alternative material for low cost blood sensors. The testing principle was based on the agglutination of red blood cells upon interaction with immunoglobin M antibodies. By using paper as the substrate, the wicking ability and transport of sample could be used as an indicator of agglutination. This paper based sensor can be applied in the developing world since the production cost is only a few cents per test. Another demonstration of utilising paper-based sensors for the analysis of blood samples was performed by Yang *et al.* [70].

Infectious bacterial agents have always been a serious problem for animals and humans. Common bacteria such as *Pseudomonas aeruginosa* and *Staphylococcus aureus* infect damaged tissue or even compromise the immune system which could lead to life-threatening diseases such as necrotising pneumonia [71]. A simple and fast sensor for detecting these bacteria is highly important considering current techniques require trained personnel to operate. Li *et al.* [63] developed a paper-based testing disc for multiplex whole cell bacteria analysis. The lateral flow immunoassay disc was made of a nitrocellulose membrane that attached to a circular plastic support. The detection of whole cell bacteria upon introduction to the membrane could be performed by analysing the change in colour intensity as the bacterial cell attached to the specific antibodies labelled with gold nanoparticles. Such a simple technique allows rapid, reagent-free analysis and direct detection of whole cell bacteria with a range of 500–5,000 CFU·mL^−1^.

### 4.2. Food Quality Control

Although most of the research efforts on paper-based devices are focused on minimising fabrication steps and overall cost for the developing world and clinical POC applications, paper has also been applied for food quality control. In the food industry, food quality control from the production process until the packaging stage is very important before a product can enter the market. Moreover, monitoring the lifetime of a product once it reaches the shelf is equally important.

Nie *et al.* [25] demonstrated the potential for ethanol analysis in food by using an electrochemical paper sensing device integrated with a commercial glucometer. The glucometer as an electrochemical reader allows on-site detection of ethanol without the need for analysis back in the laboratory. The sensor was based on the enzymatic detection of ethanol with alcohol dehydrogenase in the presence of β-NAD^+^ and ferricyanide as a mediator for electron transfer.

Other studies in food monitoring include the detection of pesticides in beverage and food samples by Hossain *et al.* [67]. The sensor consisted of acetylcholinesterase enzyme and indophenyl acetate reagent for the assay where the combination of the two created a blue colour. The presence of pesticides was indicated by a decrease in the blue colour intensity which was interpreted using a digital camera. Although the analysis still requires additional processing steps using a computer, the sensor is still considered simple and rapid compared to other sophisticated laboratory instrumentation.

### 4.3. Environmental Monitoring

In environmental monitoring, real-time detection of heavy metals and other pollutants is important as sample conditions can fluctuate over the day and also over the time course of collection and transportation back to the lab. Thus, there is a real need for sensors which can allow on-site detection and accurate monitoring of environmental conditions.

As demonstrated by Apilux *et al.* [30] a fast, simple and portable dual electrochemical/colorimetric paper-based system was developed for detecting gold and iron. The recovery of gold in industrial wastes is important as it has a high economic value, whereas iron is a common interferent in its analysis. Gold was detected down to 1 ppm by square wave voltammetry in a dilute aqua regia solution, whereas iron which interferes electrochemically with its analysis was monitored colorimetrically.

Nie *et al.* [26] created a paper-based electrochemical sensing device for the detection of Pb(II). The challenge of whether to use a single drop of solution or a stirred solution to accumulate the heavy metal was addressed by introducing a cellulose blotting pad as a sink for the outlet. This allowed continuous wicking of the solution across the electrodes which increased the efficiency and sensitivity of Pb(II) deposition during anodic stripping voltammetry.

Water contamination is a serious global problem which not only affects humans but also the ecosystem. One dangerous toxin is microcystin-LR which is produced by cyanobacteria and leads to water pollution and mass poisoning. There are many methods in detecting contamination or toxins in water, however, finding a simple, inexpensive and rapid method remains a challenge. Based on the World Health Organization (WHO), the maximum allowable concentration for microcystins in drinking water is set at 1 ng·mL^−1^ [72]. Wang *et al.* [66] reported a single-walled carbon nanotube-based paper sensor for the detection of such a toxin. The carbon nanotube-based paper was constructed by immersing the paper into a solution containing the carbon nanotubes, polymer binder and antibodies specific to the microcystin-LR. A three-electrode system was employed where the carbon nanotube-based paper was used as a working electrode to monitor the *i-t* characteristics. It was shown that microcystin-LR resulted in a decrease in conductivity of the single-walled carbon nanotubes due to alteration of the charge transfer distance between neighbouring nanotubes. The analysis technique was highly sensitive with a limit of detection 0.6 ng·mL^−1^ and was less time consuming compared to traditional methods such as ELISA (enzyme-linked immunosorbent assay).

## 5. Challenges in Paper-Based Sensors and Future Outlooks

Although there is enormous potential in paper as a platform for LOC devices, current improvements in fabrication and analysis techniques are still required in order to match the performance of conventional analytical techniques. The overall sensitivity of the device is one frequently encountered limitation. This is typically due to how the sample is introduced onto the paper, which can be at a considerable distance away from the detection zone. As the analyte is delivered across the paper, the local analyte concentration may decrease as a result of solution spreading and may also evaporate if the distance and time of travel is far from the point of introduction. The evaporation problem can be addressed by encapsulating the paper or the peripheral edge of paper using sticky tape or by placing the porous paper substrate between vinyl and polyester plastic films [30].

Furthermore, methods need to be adapted that allow the analyte to be pre-concentrated at the surface. As paper is a porous support medium, this needs to be exploited such that the cellulose fibre can accommodate a large concentration of the active species. The future should see a growth in the direct immobilisation of capture antibodies, biomolecules and nanomaterials. Ngo *et al.* [73] have reviewed attachment methods of gold, silver and titania nanoparticles to paper where they describe techniques such as wet-end addition and surface treatment. In the wet-end addition method, the nanoparticles are adhered to individual fibres during pulp processing. This technique is commonly used to prepare titania photocatalytic paper. Surface treatments can also be performed and although there are a number of treatment strategies, these generally involve the introduction of charged groups into the cellulose fibre itself followed by the use of polyelectrolyte-capped nanoparticles. As there remains a plethora of unexplored materials that can potentially be patterned onto paper, these techniques show great promise for attaching other types of molecules through electrostatic interactions.

Moreover, as paper becomes increasingly recognised as a substrate material in the research field, there will be renewed competition between manufacturers for paper types with added functionality. This could expand existing areas of research, such as sensors based on paper electronics [74] where the incorporation of the sensing material onto a relatively smooth and non-adsorbing substrate is important. Furthermore, there could be growth in paper-based piezoelectric force sensors which are traditionally constructed from silicon-based materials. Recently, Liu *et al.* [75] developed a paper-based MEMS (Micro-Electro-Mechanical System) weighing balance. The realisation of such a device can potentially open up a whole new division within paper sensing. By readapting existing fabrication methods and analysis techniques onto paper using approaches which at first glance, appear unfeasible, there could be a rise in more advanced sensors, as well the incorporation of more dual-based detection schemes for more accurate monitoring in a variety of sample media.

Another challenge faced by researchers is the difficulty in multiplex analysis. Thus far, there have been some innovative developments based on paper-cut microfluidic devices [12] and 3-D paper devices [19,53] which were discussed in earlier sections. However, as the shape and design becomes more complex, there is the potential for cross-talk as some of the signal reporters may diffuse to neighbouring channels. Efforts by *Ge et al.* have been made to reduce cross-talk through the way in which the washing procedure was carried out [50]. Furthermore, care must be considered in the spotting of reagents and the importance of microfluidic pathways and the location for analysis. The future for multiplex analysis could see the development of more intricate 3-D paper architectures that can detect multiple analytes using a combination of techniques.

Finally, there is the challenge of utilising analysis techniques that meet the requirements of low power and portability. For colorimetric assays there has been a move towards the use of camera phones to interpret the result [1,12,22,23,57]. Electrochemical techniques have been developed into hand-held readout devices, such as the glucometer, which also provides the electrochemical measurement [26]. However, in order for a device to be truly low power and low cost, the minimisation of extra components, where possible, is necessary. One such innovation is described by Thom *et al.* [76] where fluidic batteries were used as a power source in paper-based microfluidic devices. The galvanic cells were integrated within the microfluidic channel of the paper which would turn on upon sample introduction. Thus, the sample was used to generate power for conducting an assay. This very attractive idea could see its usage in a resource-limited environment and the utility of a power source for optical readout assays and electrochemical detection schemes.

With advancing methodologies, there could be scope for new application areas, as portability options become more viable and with the realisation that low cost paper-based devices can meet the objectives for sensitive and accurate monitoring. The detection of chemical warfare agents, and monitoring of drugs of abuse are just a few examples of application areas where paper-based sensors may be suitable.

Following the review on existing paper-based sensors, it is clear that extensive development has been made in the last few years that have led to “smarter” paper-based sensors and there is no doubt that the future would see more advances in this growing research field. Further research related to fabrication techniques and the incorporation of functional materials onto the surface need to be considered in order to overcome the challenges discussed above, in order to produce better, more stable devices capable of measuring multiple analytes at a high sensitivity. It will be an important challenge not to sacrifice the simplicity and cost advantages of paper-based sensors in order to achieve such devices.

## Figures and Tables

**Table 1.** Comparison of different analysis methods.

**Detection Method**	**Reference**	**Quantitative/Qualitative?**
Colorimetric	Martinez *et al.* [11], Wang *et al.* [12], Fenton *et al.* [13], Dungchai *et al.* [59].	Semi-quantitative
	Martinez *et al.* [22], Klasner *et al.* [20], Abe *et al.* [23], Ratnarathorn *et al.* [57].	Quantitative
Electrochemical	Dungchai *et al.* [5, 24], Nie *et al.* [25,26], Lankelma *et al.* [58], Shiroma *et al.* [18], Apilux *et al.* [30], Liu *et al.* [55], Carvalhal *et al.* [16].	Quantitative
Electrical conductivity	Steffens *et al.* [29]	Semi-quantitative
	Arena *et al.* [28]	Quantitative
Chemiluminescence and electrochemiluminescence	Yu *et al.* [7], Delaney *et al.* [27], Ge *et al.* [50,53].	Quantitative
